# Prioritising Age‐Friendly Care Decreases the Use of Physical Restraints in Patients with Dementia and Delirium in the Acute Hospital

**DOI:** 10.1002/alz70858_102698

**Published:** 2025-12-25

**Authors:** Jing Wei Lim, Vanessa Lunardi, Suhui Lee, Wen Qi Mok, Jessie Joon Cheen Tan, Aloysius Jun An Soh, Qian Ci Tang, Thuy‐Anh Giang, Philip Lin Kiat Yap

**Affiliations:** ^1^ Khoo Teck Puat Hospital, Singapore, Singapore

## Abstract

**Background:**

The Department of Geriatric Medicine at Khoo Teck Puat Hospital provides an interdepartmental referral service for patients admitted with cognitive and behavioural issues termed 6VS. 6VS refers to cognition as the 6^th^ Vital Sign, but also represents the 6 Care Vitals for the Vulnerable Senior which references our 6M framework of age‐friendly care (what Matters, Mobility, Mentation, Medication, Multimorbidity and Malnutrition). The 6VS service also incorporates Humanitude care methodology in reducing hospital‐acquired harm.

**Methods:**

Data of 670 patients referred to 6VS service (81.8±8.4 years, 54% female, CFS 5.72±0.9) from January to December 2024 was analysed and categorised by wards (geriatric [*n* = 397], non‐geriatric [*n* = 255], and private wards [*n* = 18]). Patient profiles, difficulties in care, and use of physical restraints were examined using descriptive statistics. Top 3 difficulties in care were identified by analysing most common key phrases found in 627 patient records.

**Results:**

Patient profiles of referrals from geriatric and non‐geriatric wards were analysed. There were a total of 397 geriatric ward patients (86.2±5.6 years, 53% female, CFS 5.74±1.0) and 255 non‐geriatric ward patients (74.8±7.5 years, 54% female, CFS 5.65±0.8). Activity engagement, poor oral intake, and agitation are the top 3 most common difficulties in care for geriatric and non‐geriatric ward patients, albeit reported in different proportions (activity engagement: 47% geriatric, 18% non‐geriatric; poor oral intake: 21% geriatric, 16% non‐geriatric; agitation: 18% geriatric, 46% non‐geriatric). A total of 95 restraints (5 geriatric, 84 non‐geriatric, 6 private) were reported between January to December 2024, out of which 62 restraints were removed (5 geriatric, 51 non‐geriatric, 6 private).

**Conclusion:**

Prioritising the 6M framework is fundamental to care in our geriatric wards, which has translated to a decreased reliance on physical restraints. In addition, geriatric intervention in non‐geriatric care settings demonstrated that a large proportion of physical restraints in non‐geriatric care settings can be removed. A focus on patient‐centred age‐friendly interventions improves quality of care for patients with dementia/delirium in an acute hospital.

